# High‐Frequency Ultrasound Boosts Bull and Human Sperm Motility

**DOI:** 10.1002/advs.202104362

**Published:** 2022-02-09

**Authors:** Junyang Gai, Esma Dervisevic, Citsabehsan Devendran, Victor J. Cadarso, Moira K. O'Bryan, Reza Nosrati, Adrian Neild

**Affiliations:** ^1^ Department of Mechanical and Aerospace Engineering Monash University Clayton Victoria 3800 Australia; ^2^ School of BioSciences Faculty of Science the University of Melbourne Parkville Victoria 3010 Australia

**Keywords:** acoustofluidics, infertility, male infertility, microfluidics, sperm, surface acoustic wave

## Abstract

Sperm motility is a significant predictor of male fertility potential and is directly linked to fertilization success in both natural and some forms of assisted reproduction. Sperm motility can be impaired by both genetic and environmental factors, with asthenozoospermia being a common clinical presentation. Moreover, in the setting of assisted reproductive technology clinics, there is a distinct absence of effective and noninvasive technology to increase sperm motility without detriment to the sperm cells. Here, a new method is presented to boost sperm motility by increasing the intracellular rate of metabolic activity using high frequency ultrasound. An increase of 34% in curvilinear velocity (VCL), 10% in linearity, and 32% in the number of motile sperm cells is shown by rendering immotile sperm motile, after just 20 s exposure. A similar effect with an increase of 15% in VCL treating human sperm with the same setting is also identified. This cell level mechanotherapy approach causes no significant change in cell viability or DNA fragmentation index, and, as such, has the potential to be applied to encourage natural fertilization or less invasive treatment choices such as in vitro fertilization rather than intracytoplasmic injection.

## Graphical abstract

Sperm motility plays a central role in both natural fertilization and clinical sperm selection as the key male fertility parameter. However, there is an absence of methods to improve sperm motility without introducing more damage. Here, it is found that treating sperm cells with high frequency ultrasound results in a boost in sperm motility by enhancing sperm metabolism.

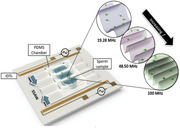

## 1 Introduction

Infertility is a rising global health issue that impacts over 70 million couples per year.^[^ ^1^ ^]^ Male factor infertility accounts for 50% of infertility cases, primarily mediated by deficits in sperm number and/or function.^[^ ^2^, ^3^, ^4^ ^]^ Sperm motility plays a central role in both natural fertilization and clinical sperm selection as the key parameter reflecting the fertility potential of individual sperm.^[^ ^5^, ^6^ ^]^ In natural reproduction, sperm must traverse a complex route from the seminal fluid to the viscous fluids contained within the cervix, uterus, the fallopian tube, and ultimately through the highly folded and complex lumens within the tube to achieve fertilization.^[^ ^7^, ^8^ ^]^ However, sperm motility can be impaired through factors attributed to the modern lifestyle, such as pollution and unhealthy diets,^[^ ^1^ ^]^ or through endogenous genetic factors.^[^ ^9^ ^]^ To circumvent infertility problems, assisted reproductive technologies (ART) have been developed.^[^ ^10^ ^]^ For assisted reproduction, motile sperm are collected via two main clinical methods; the swim up approach,^[^ ^11^ ^]^ in which sperm swim out of a sedimented raw semen sample into a physiological buffer, or through the density gradient centrifugation method,^[^ ^7^ ^]^ where motile sperm are indirectly selected via centrifugation through a density gradient. The relatively long duration of these clinical selection processes, increases the degree of oxidative stresses in the sample,^[^ ^12^ ^]^ which may in turn reduce sperm motility levels and increase DNA fragmentation.^[^ ^13^ ^]^ There is an absence of noninvasive, drug‐free methods, to restore or enhance sperm motility without introducing potential damage to their viability or DNA integrity.

In humans, the sperm tail or flagella is ≈60 µm in length and is composed of a relatively rigid midpiece which contains the mitochondria, attached to a more flexible principal piece and at the extremity an end piece wherein the axoneme, the driveshaft of the sperm tail, is surrounded solely by the plasma membrane.^[^ ^14^ ^]^ A sperm's energy for normal cellular function and motility is mainly synthesized via two distinct mechanisms, through glycolysis, which occurs in the head and the principal piece of flagellum, and in the mitochondria through an oxidative phosphorylation process. In both of the processes, adenosine triphosphate (ATP) is produced and used to drive the ATP‐activated dynein motors within the axoneme.^[^ ^15^ ^]^ The ATP‐activated dynein arms sequentially slide one of the nine outer microtubules in the 9 + 2 axoneme structure over the neighboring doublet to generate the flagellar waveform, where dynein‐tubulin binding affinity is regulated by long‐residence ATP binding site.^[^ ^16^ ^]^ Changes in sperm motility are likely linked to an increased mitochondrial respiration as observed in several species during capacitation and hyperactivation.^[^ ^17^ ^]^ Indeed, during a sperm's metabolic process, up to 2% of the intracellular oxygen (O_2_) is converted into superoxide radicals (O_2_^¯^) followed by the production of hydrogen peroxide (H_2_O_2_) and hydroxyl radicals (OH^¯^), both types of reactive oxygen species (ROS).^[^ ^17^ ^]^ ROS molecules are required for sperm capacitation^[^ ^18^ ^]^, the acrosome reaction^[^ ^19^ ^]^ and sperm‐oocyte binding,^[^ ^20^ ^]^ which in turn are required for fertilization. Since unbalanced and high ROS concentrations can lead to lipid peroxidation, protein inactivation, and nucleotide damage, the intercellular ROS concentration is usually maintained at physiological levels by means of intrinsic and external antioxidant mechanisms to ensure cell integrity.^[^ ^19^ ^]^ The molecular mechanisms of intercellular ROS management in sperm are yet to be fully understood, however, they play a vital role in redox signaling and oxidative damage that influence sperm motility.^[^ ^20^, ^21^ ^]^

Regulation of cell metabolism has been of great interest in many therapeutic applications such as cancer treatment,^[^ ^22^ ^]^ injury healing,^[^ ^23^ ^]^ and drug metabolism.^[^ ^24^ ^]^ Mechanotherapy, in particular, has been applied as a noninvasive approach in tissue engineering to alter, or improve, the metabolic function of a target cell.^[^ ^25^ ^]^ In mechanotherapy, an applied physical stimulus is employed to promote tissue regeneration by exploiting a series of biochemical reactions within the cell.^[^ ^25^ ^]^ Mechanotherapy methods such as microdeformational wound therapy,^[^ ^26^ ^]^ shock wave therapy,^[^ ^27^ ^]^ and nanovibration enhanced osteogenesis^[^ ^28^ ^]^ have been successfully applied to relieve symptoms and promote rehabilitations toward predamaged or presurgical levels. However, despite its potential to alter the metabolic function of a target cell at the single cell level,^[^ ^29^ ^]^ mechanotherapy is yet to be applied as a direct treatment method for a specific disease.

A common approach that has gained popularity recently is acoustic‐based mechanotherapy, where bulk acoustic waves (BAW) with the frequency ranging from 0.1–5 MHz cause an intentional pressure disturbance within a tissue^[^ ^30^ ^]^ to improve blood circulation,^[^ ^31^ ^]^ and reduce cell death by either necrosis^[^ ^32^ ^]^ or apoptosis.^[^ ^33^ ^]^ In contrast to BAW, surface acoustic waves (SAW) are typically used for operation at a higher frequency (typically 20—350 MHz) to achieve a higher precision in single cell level manipulation^[^ ^34^ ^]^ for sorting,^[^ ^35^, ^36^ ^]^ patterning,^[^ ^37^ ^]^ capturing,^[^ ^38^ ^]^ and sonoporation^[^ ^39^ ^]^ of live cells, as SAW wavelength is in the same range as typical cells’ sizes. This capability points to a potential for higher accuracy and efficiency in mechanotherapy‐based applications. Indeed, Devendran et al.^[^ ^40^ ^]^ recently reported a consistent increase of metabolic activity for adherent, nonmotile cells post‐SAW exposure, suggesting the potential utilization of this method for single cell level mechanotherapy.

Assisted reproductive technology contains a graduation of treatment depending on the severity of sperm dysfunction in the semen sample.^[^ ^11^ ^]^ Typically intrauterine insemination is being used as the treatment method for men with mild sperm defects, whilst in vitro fertilization (IVF) and intracytoplasmic injection (ICSI) are being used for moderate and severe infertility cases, respectively.^[^ ^10^ ^]^ In many cases, prior to ICSI or even IVF, chemical stimulation after physically removing sperm seminal plasma is being adopted to improve sperm motility and detect viability.^[^ ^41^ ^]^ However, these methods face the limitations as they require long incubation period (≈15 min)^[^ ^42^ ^]^ and are based on extensive washing procedures (≈1 h).^[^ ^42^ ^]^ Here, we present a new method to boost sperm motility by increasing the intercellular rate of metabolic activity using SAW. By studying the relationships between sperm velocity and acoustic parameters, we reveal that our acoustic‐based method significantly improves the motility characteristics of sperm by 30% after a brief 20 s acoustic exposure at a frequency of 19.28 MHz and power of 2 W. Compared with current clinical methods to increase sperm motility, this high‐frequency acoustic‐based method is biocompatible, without any adverse effects on either sperm viability or DNA integrity. Moreover, ultrasound has been utilized prevalently in different medical applications including diagnostics, therapy, and surgery,^[^ ^43^ ^]^ without noticeable side‐effect, which indicates the noninvasiveness of this approach. Cells exposed to high frequency ultrasound demonstrate a higher rate of metabolic activity, producing more energy required for swimming, and its ability to swim faster by beating at higher frequencies and with larger amplitudes. This single cell level mechanotherapy approach provides a new opportunity to boost sperm motility in assisted reproduction, particularly for samples that suffer from poor sperm motility, hence improving the outcome of assisted reproductive technology.

## 2 Results

**Figure 1:**
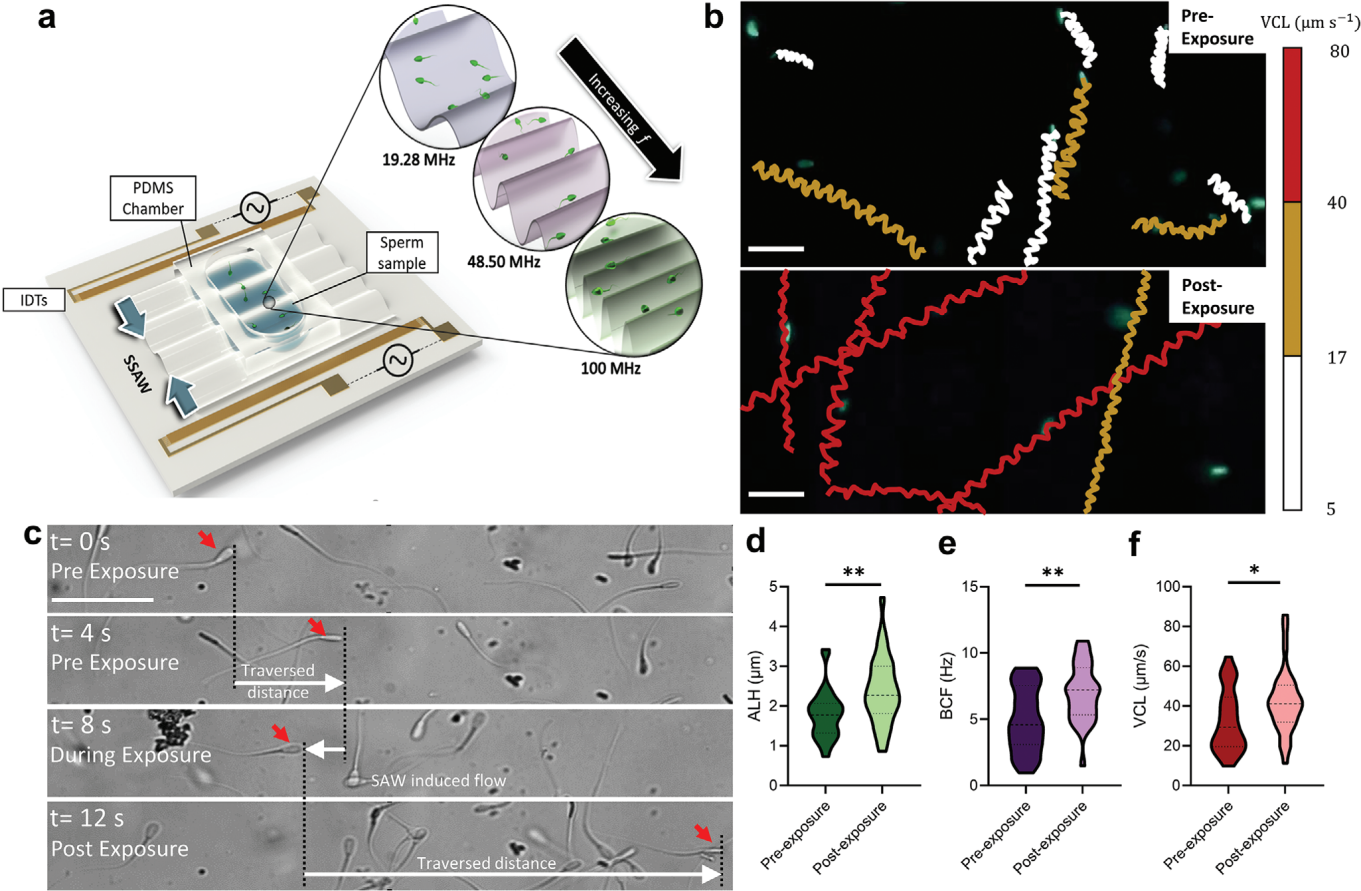
Sperm motility boost post‐acoustic actuation. a) As shown schematically, interdigital transducers (IDTs) are used to generate standing surface acoustic waves (SSAW) in the chamber containing the sperm sample. b) Time‐lapsed images (see Movie S1, Supporting Information) demonstrate the distance traveled by an individual sperm pre‐, during, and post‐exposure (ultrasound applied at *t* = 4 s). Red arrows specify the tracked sperm cell. c) Representative sperm swimming trajectories pre‐ and post‐exposure to ultrasound indicate the higher curvilinear velocity (VCL) of sperm post‐ultrasound exposure (19.28 MHz, 2 W, 20 s). The color corresponds to curvilinear velocity amplitude. Scale bars, 50 µm. d) A comparison of sperm lateral head displacement (ALH), e) beat cross frequency (BCF), and f) curvilinear velocity (VCL) pre‐ and post‐exposure show a significant increase in both metrics post‐exposure. All data represented as mean ± s.d. from three independent experiments (*n* = 10 sperm per experiment). *P*‐values were determined by the two‐tailed unpaired *t*‐test, * *p* ≤ 0.05, ** *p* ≤ 0.01.

**Figure 2:**
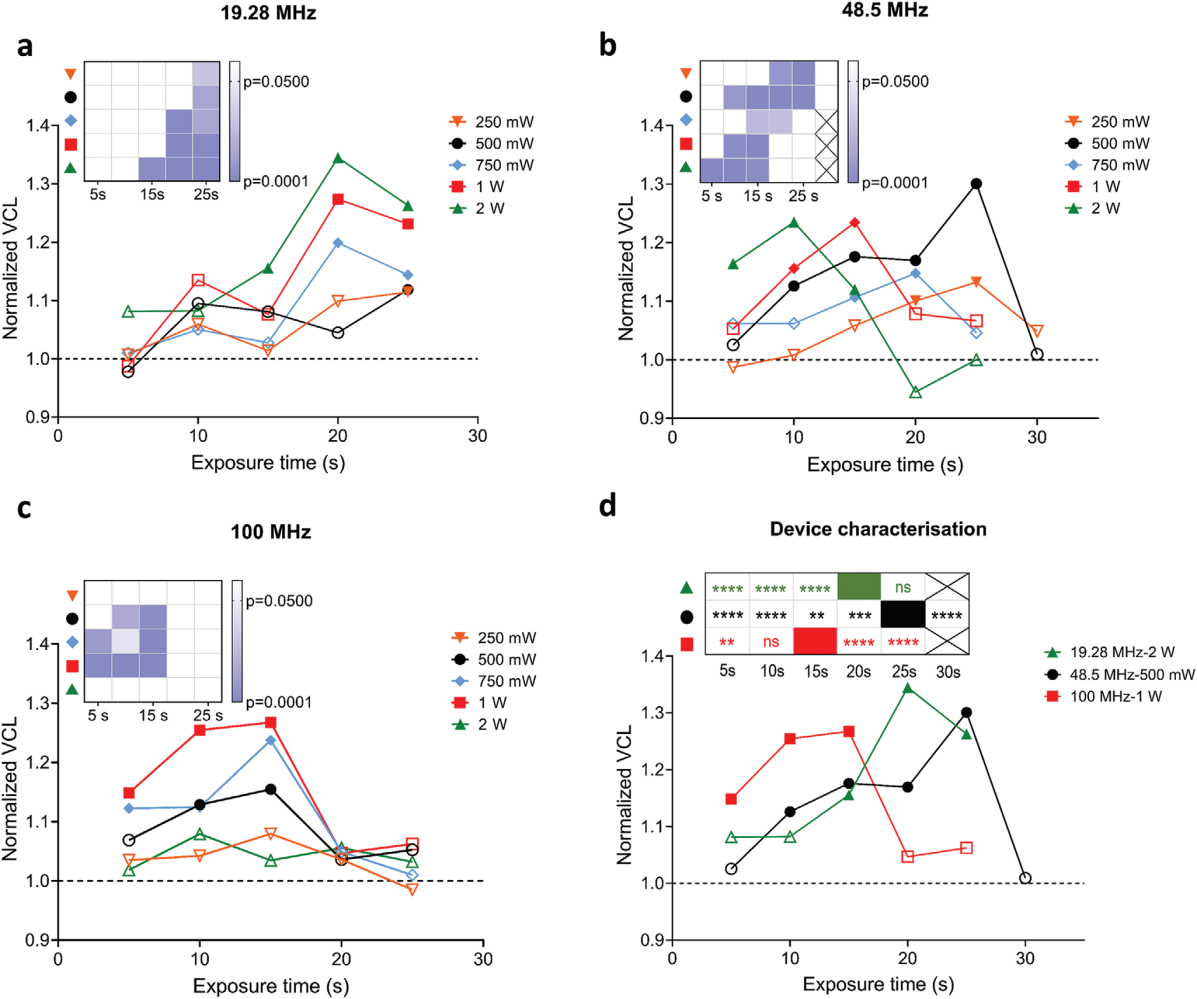
Increase in sperm curvilinear velocity (VCL) post‐exposure to ultrasound. Effect of power and exposure time at an SAW frequency of a) 19.28 MHz, b) 48.5 MHz, and c) 100 MHz on the group‐mean centered VCL post‐exposure. d) Relative resultant normalized post‐exposure VCL as a function of exposure time when excited at frequencies of 19.28, 48.5, and 100 MHz (at optimum excitation powers). The panels inset in (a)–(c) show *p*‐values of statistical analysis of group‐mean centered post‐exposure VCL in comparison with pre‐exposure VCL at each exposure condition with hollow‐shaped data point representing statistically nonsignificant (*p* > 0.05) and solid‐shaped data point representing statistically significant (*p* ≤ 0.05) data when excited at frequencies at 19.28, 48.5, and 100 MHz. The panel inset in (d) shows *p*‐values resulting from the statistical analysis of the performance of other exposure times in comparison with the performance of the exposure condition where the highest change was achieved. All data represented as grand mean from three independent experiments performed using different samples from three different bulls. (>40 cells per bull). Sperm trajectories are recorded for the same sample in the same imaging field pre‐ and post‐exposure. All *p*‐values were determined using the two‐way ANOVA with post hoc Bonferroni corrections, * *p* ≤ 0.05, ** *p* ≤ 0.01, *** *p* ≤ 0.001, **** *p* ≤ 0.0001, and ns represents no significance.

An acoustofluidic platform was used to probe the effects of acoustic exposure on sperm motility. The device comprised of an SAW chip and a polydimethylsiloxane (PDMS) microfluidic chamber, within which the sperm sample was introduced (**Figure** **1**a). A pair of interdigitated transducers (IDTs) were used to generate counter‐propagating waves along a substrate upon excitation of an AC electric field.^[^ ^44^, ^45^, ^46^ ^]^ This energy couples from the substrate into fluid placed upon it, offering an efficient method of generating high‐frequency sound fields for accurate cell manipulation,^[^ ^47^, ^48^ ^]^or, in this case, to expose sperm cells to a pre‐defined ultrasonic stimulation. Figure 1b depicts the swimming trajectories of sperm pre‐ and post‐exposure, with the color of trajectories corresponding to the average swimming velocity of sperm along its instantaneous trajectory (average curvilinear velocity, VCL). It is noteworthy that, while the flow generated due to ultrasound exposure diminishes immediately with the interruption of ultrasound exposure, the swimming velocity of sperm after exposure remains considerably higher than their pre‐exposure velocity (**Figure** **2**b,c and Movie S1, Supporting Information). Over a range of exposure power and frequency assessed (Figure 2), ultrasonic exposure at 19.28 MHz and 2 W was observed to maximally increase the distance traveled by an individual sperm per unit time (Figure 1c)–an effect which persists post‐exposure. This optimal setting resulted in a 34% increase in sperm swimming velocity. The increase in the swimming velocity post‐exposure is related to an increase in both lateral head displacement (ALH) and beat cross frequency (BCF), indicating that acoustic exposure influences the fundamental characteristics of sperm beating behavior (Figure 1d,e). Specifically, ALH increased by 34% (*n* = 30, *N* = 3, *p *= 0.0006) and BCF increased by 42% (*n* = 30, *N* = 3, *p* < 0.0001) post‐exposure. Several factors might contribute to regulate the sperm motility behavior, including an increase in the rate of metabolic activity,^[^ ^40^ ^]^ regulation of mechanosensitive ion channels that influences the flagellar waveform,^[^ ^49^ ^]^ and a change in the stiffness of the cell membrane that can influence the rate of energy dissipation into the fluid.^[^ ^50^ ^]^

The pre‐ and post‐exposure sperm VCL have been examined over a range of ultrasonic excitation conditions. Independent experiments using different cryopreserved bull semen samples (>40 sperm examined for each sample) were conducted for each exposure condition. The significance of the difference between pre‐ and post‐exposure for an individual exposure condition and the relative increase in motility over the range of conditions was examined. After the samples were exposed to ultrasound, the VCL was measured for each sperm detected in the same imaging field and this value was normalized to the pre‐exposure group mean (see Tables S1–S3, Supporting Information); for example, a normalized VCL of 1.3 represents an average increase of 30% in the VCL measured post‐exposure compared to pre‐exposure for that exposure condition. The normalized VCL was quantified for a range of ultrasonic frequencies (19.28, 48.5, and 100 MHz), powers (250 mW to 2 W), and exposure times (5–25 s), as plotted in Figure 2a–c. Figure 2a shows the normalized VCL for 19.28 MHz over a range of exposure times (5–25 s) and powers (250 mW to 2 W). For 20 s of exposure at 2 W, the maximum increase (34%) in sperm VCL was achieved (*p* < 0.05 when compared with the corresponding pre‐exposure VCL, Figure 2a). In contrast, shorter exposure times at this power level were not sufficient to generate a stable and consistent increase in sperm VCL, reflected by the statistically insignificant result (shaded regions in panel insets in Figure 2, see Tables S4–S6, Supporting Information). For 19.28 MHz, there is a lower cutoff in exposure time of 15 s for 2 W of power, below which the VCL change is not significant, perhaps unsurprisingly given that the dosage is a function of both exposure time and power. Therefore, this cutoff occurs at a longer exposure time for lower powers. For the case of 48.5 MHz and 2 W, an upper limit of 15 s to the exposure range is also observed beyond which the VCL change loses significance. As with the lower exposure time limit, this upper exposure time limit is larger at lower powers.

In Figure 2d, the frequency versus operating power conditions which resulted in the highest increase in the post‐exposure VCL was measured. For each of these operating conditions, the significance across the range of exposure times is shown, thereby allowing the selection of the optimum excitation conditions. The highest VCL change of 34% was achieved for the exposure condition of 19.28 MHz and 2 W with an exposure time of 20 or 25 s (no statistically significant difference). For 48.5 MHz exposure at 500 mW, an exposure of 20 s resulted in a VCL increase of 30% which was statistically higher than both shorter and longer exposure times.

**Figure 3:**
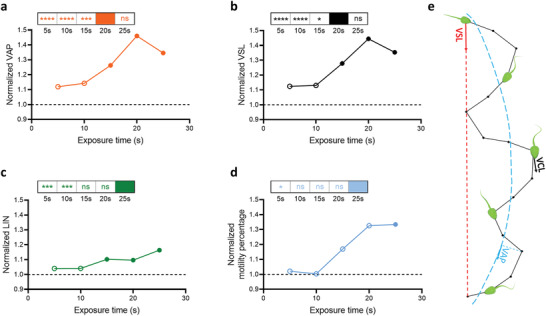
Effect of ultrasonic exposure time on sperm motility parameters when excited at a frequency of 19.28 MHz, 2 W. Relative resultant normalized post‐exposure a) average path velocity (VAP), b) straight line velocity (VSL), c) linearity (LIN), and d) proportion of motile sperm. Solid data points indicate a significant difference between pre‐ and post‐exposure, whilst outlined markers indicate statistically nonsignificant results (*p* > 0.05). The panel insets show *p*‐values resulting from the statistical analysis of the performance of other exposure times in comparison with the performance of the exposure condition where the highest change was achieved. All data represented as grand mean from nine independent experiments performed using triplicate samples (three data sets per bull and three different bulls; *n* = 9, >40 cells per bull). *p*‐values were determined using two‐way ANOVA test with post hoc Bonferroni corrections. * *p* ≤ 0.05, ** *p* ≤ 0.01, *** *p* ≤ 0.001, **** *p* ≤ 0.0001, and ns represents no significance.

To further characterize the effect of acoustic excitation to boost sperm motility, a range of additional parameters pre‐ and post‐exposure were quantified. The quantified sperm motility parameters include the average path velocity (VAP: swimming velocity along the time‐averaged trajectory, **Figure** **3**a), straight line velocity (VSL: time average velocity along the straight line between the start and final tracking points, Figure 3b), linearity (LIN = VSL/VCL; Figure 3c), and motility percentage (Figure 3d). Figure 3 details these sperm motility parameters contrasting pre‐ and post‐exposure using a frequency of 19.28 MHz and power of 2 W. After 20 s of exposure (the optimum exposure time based on probability distribution functions of VCL, Figure S2, Supporting Information), the sperm swim both faster and straighter, exhibiting 46% increase in VAP and 44% increase in VSL. It is noteworthy that the percentage increase of both VAP and VSL were higher than VCL, mainly due to a 10% increase in LIN. The results indicate that ultrasonic exposed sperm exhibit both an increase in instantaneous swimming velocity and swim along a straighter path (as was also indicated by in Figure 1c). Such straighter motion has previously been linked to a decrease in the level of bend asymmetry in the flagellar wave.^[^ ^8^ ^]^ Furthermore, the percentage of motile sperm increased by 33% for the longest exposure time of 25 s (Figure 3d). This increase in the number of motile sperm post‐exposure was attributed to ultrasonic exposure rendering immotile sperm, motile (see Movie S2, Supporting Information). As sperm motility is a significant predictor of male fertility potential and is directly linked to fertilization success,^[^ ^51^ ^]^ the “recruitment” of additional motile sperm after ultrasonic exposure would predict increased success in in vivo and in vitro fertilization.

**Figure 4:**
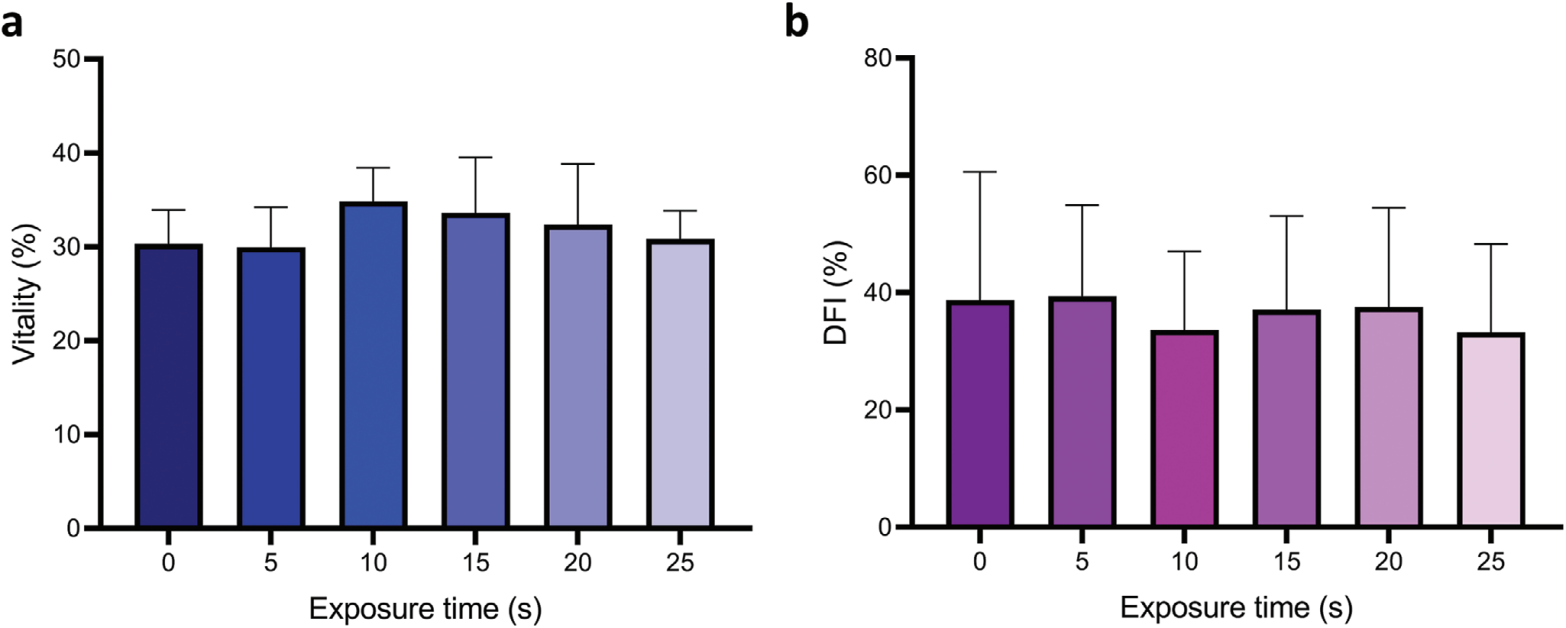
Bio‐compatibility assessment post‐ultrasonic exposure at the frequency of 19.28 MHz, 2 W. Changes in a) sperm viability and b) DNA fragmentation index (%DFI) as a function of exposure time. All data represented as mean ± s.d. from three independent experiments from three different bulls. No significant difference is observed for all data sets based on *p*‐values that were determined using one‐way ANOVA test.

To test the biocompatibility of our method, sperm vitality and DNA integrity pre‐ and post‐ultrasonic exposure (19.28 MHz, 2 W) were compared (**Figure** **4**). No significant changes in sperm live/dead ratio were observed as shown in Figure 4a, indicating that the cells preserve their membrane integrity even after the 20 s of acoustic exposure required to achieve the highest relative increase in VCL (Figure 2). Similarly, the DNA fragmentation index (DFI, indicating the percentage of sperm with fragmented DNA) remained unchanged by ultrasonic exposure, demonstrating that the treatment does not have an untoward impact on interrupting sperm intracellular DNA structures or increasing DNA damage level.

## 3 Discussion

Overall, the results indicate that the average VCL of a sample of sperm can be increased by ultrasound when using a suitable combination of frequency, power, and exposure time. Whilst low acoustic energy may be insufficient to generate a statistically effective VCL boost, if the exposure is too prolonged it may have a negative effect. The deleterious nature of over exposure could potentially be due to the induced mechanical stresses. Excessive stresses cause increased mitochondrial membrane permeability and interrupted ATP production^[^ ^52^ ^]^ and thus compromised sperm motility.^[^ ^53^ ^]^ Moreover, prolonged mechanical stimulation has been shown to increase membrane stiffness in adherent cells^[^ ^50^ ^]^ and reduce membrane fluidity and increase cell rigidity has been shown to contribute to reduced sperm motility levels under other conditions.^[^ ^54^ ^]^

**Figure 5:**
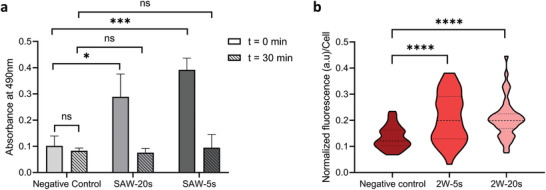
Analysis for metabolic activity and intracellular ROS changes post‐ultrasonic exposure. a) Metabolic activity changes in bull sperm cells without exposure (Negative control) and post‐exposure to ultrasound for 20 and 5 s at 19.28 MHz, 2 W, with the assay initiated immediately (*t* = 0 min) and 30 min pos‐tultrasound treatment (*t* = 30 min). b) The average fluorescence intensity (arbitrary counts, a.u.) as a measure of H_2_O_2_ concentration of sperm cells without exposure (Negative control) and postexposure to ultrasound with the same parameters as (a). All data represented as mean ± s.d. from four independent experiments with >45 sperm analyzed per experiment. *p*‐values were determined by one‐way ANOVA test with Bonferroni corrections. * *p* ≤ 0.05, *** *p* ≤ 0.001, **** *p* ≤ 0.0001, and ns represents no significance.

Our results also demonstrate the biocompatibility of this method by showing no significant changes on sperm viability and DNA integrity (**Figure** **5**). Sperm DNA integrity is an important indicator of male infertility, and strongly correlated with the success rate of natural repoduction and the outcome of assisted reproduction.^[^ ^55^ ^]^ Combined with the significant effect of enhanced sperm motility and the lack of adverse effects associated with biocompatibility imposed by ultrasonic exposure, our results demonstrate the significant potential to boost sperm motility, benefitting patients with male infertility issues in assisted reproduction.

Sperm motility is produced by sperm flagellar beating^[^ ^7^ ^]^ and an improvement in sperm motility is associated with the rate of energy production and its utilization by the flagellum.^[^ ^56^ ^]^ ATP is produced via glycolysis or oxidative phosphorylation via the electron transportation from nicotinamide adenine dinucleotide (NADH) to oxygen via NADH dehydrogenase protein.^[^ ^57^ ^]^ ATP hydrolysis releases energy^[^ ^16^ ^]^ to drive the dynein molecular motor in the 9 + 2 structure of the flagellar axoneme.^[^ ^53^ ^]^ Not surprisingly, a change in the rate of energy production can influence sperm motility.

Our results show that high frequency (19.28 MHz) ultrasonic exposure at 2 W for 20 s boosts sperm swimming velocity without adverse effects on either their viability or DNA integrity (Figure 4). This increase in swimming performance is potentially due to an alteration in the regulation of bioenergetic mechanisms controlling flagellar activity. Flagellar bending occurs as a result of the activation of ATPases associated with diverse cellular Activities (AAA) domains on axonemal dyneins.^[^ ^14^ ^]^ At these sites, chemical energy from ATP hydrolysis is transduced to mechanical energy causing the sliding of the outer microtubule doublets,^[^ ^7^ ^]^ resulting in a rhythmic beating of the flagellum. To probe potential alterations to this process as a result of ultrasonic exposure, the rate of metabolic activity was assessed using an MTS assay [(3‐(4,5‐dimethylthiazol‐2‐yl)‐5‐(3‐carboxymethoxyphenyl)‐2‐(4‐sulfophenyl)‐2H‐tetrazolium assay] to examine the sperm metabolic rate both immediately and 30 min postexposure. Sperm cells exposed to ultrasound (19.28 MHz, 2 W) for 5 and 20 s demonstrated a significantly higher rate of metabolic activity than the negative control of unexposed sperm cells (Figure 5a). The rate of metabolic activity was higher for 30 min post‐exposure, and the VCL of exposed sperm remained significantly higher than the control group for up to 5 min post‐exposure (Figure S4, Supporting Information). This ultrasound induced increase in sperm metabolic activity acts as a mechanism to boost sperm motility. An increase in metabolic activity in sperm can be linked directly to a higher rate of energy production.^[^ ^58^ ^]^ In sperm, 0.2–2% of the O_2_ involved in the mitochondrial respiratory chain, where NADH and succinates are oxidized to generate energy for ATP synthesis, escapes, and produces ROS.^[^ ^59^ ^]^ Hence, increased metabolism inevitably results in a higher level of ROS production in the cell.^[^ ^18^ ^]^ Whilst low to moderate levels of ROS is required to regulate physiological functions,^[^ ^60^ ^]^ at high concentrations, the effects can be detrimental.^[^ ^61^ ^]^ ROS at high concentrations causes decrease in both sperm motility and DNA integrity.^[^ ^52^ ^]^ To examine this, we measured the level of H_2_O_2_ in the cells, this being a common form of endogenous ROS. A higher concentration of intracellular H_2_O_2_ was found in exposed cells (benchmarked against unexposed cells; negative control), as shown in Figure 5b. However, with increased ROS production post‐ultrasonic exposure, no significant changes were witnessed for sperm viability and DNA integrity (Figure 4), indicating that the increased level of ROS was not adequate to damage the sperm DNA and viability.

This increase in metabolism and sperm motility postultrasonic exposure might be attributed to effects on cell signaling pathways. An influx of Na^+^, H^+^, Ca^2+^ and/or ${\mathrm{HCO}}_{3}^{-}$ can cause an increase in sperm motility, via the activation of ion channels.^[^ ^62^ ^]^ In endothelial cells and neurons, ultrasonic exposure has been linked to changes in cell membrane permeabilization accompanied by an influx of Ca^2+^ through voltage gated calcium channel over a range of frequencies.^[^ ^29^, ^49^ ^]^ In sperm, Ca^2+^ influx can alter sperm flagellar waveform.^[^ ^63^ ^]^ The persistence of an increase in sperm motility post‐ultrasonic exposure in a Ca^2+^‐free buffer, however, strongly suggests that an influx of extracellular Ca^2+^ is not the underlying mechanism (see Figure S2, Supporting Information). In theory, however, the mobilization of intracellular Ca^2+^, for example stored at the base of flagellum,^[^ ^64^ ^]^ may propagate down the flagellar midpiece upon activation, and play a role in boosting motility. Previously published data show that elevation of Ca^2+^ along the flagellum increases the amplitude of the flagellar bend on one side,^[^ ^64^, ^65^, ^66^ ^]^ producing a highly asymmetrical swimming pattern, known as sperm hyperactivation,^[^ ^63^, ^67^ ^]^ suggests changes in intracellular Ca^2+^ do not underpin the current observations. Compared with a sperm's normal (activated) swimming pattern, hyperactivated sperm display increased swimming velocity and flagella amplitude, but decreased linearity and an asymmetric flagella waveform. As evidenced here exposure of noncapacitated to ultrasonic frequencies resulted in an increase in linear velocity, i.e., sperm were not induced to hyperactivate. Therefore, the motility boost effect post‐ultrasonic exposure cannot be sufficiently explained by the change in Ca^2+^ concentration, neither intracellularly nor extracellularly.

For natural reproduction, the number of motile sperm in the sample is directly linked to fertilization success,^[^ ^11^ ^]^ and in the case of assisted reproduction, the number of motile sperm in the processed sample is an important consideration when selecting the treatment method.^[^ ^68^ ^]^ For example, in IVF, a sample of over 50 000 motile sperm^[^ ^69^ ^]^ with an average velocity of over 50 µm s^−1^ can achieve the best fertilization rate as compared with samples of lower swimming velocity.^[^ ^70^ ^]^ In ICSI, as the other common treatment method, although sperm motility is not required but is being used commonly as the key criteria to select an individual sperm for the ICSI cycle,^[^ ^71^ ^]^ and in more challenging samples (such as testicular samples), inducing motility (for example via pentoxifyline) is a routine approach to detect viable sperm for ICSI injection.^[^ ^72^ ^]^ Additives such as cAMP,^[^ ^73^ ^]^ pentoxifylline,^[^ ^74^ ^]^ and platelet activating factor^[^ ^75^ ^]^ have also been used in clinics to enhance sperm motility.^[^ ^42^ ^]^ However, these methods involve labor‐intensive protocols and can be invasive,^[^ ^42^ ^]^ with potential harmful effects on embryo development.^[^ ^76^ ^]^ In comparison with these conventional methods, ultrasound offers both noninvasiveness and time efficiency, considering the achieved improvement of ≈30% in both curvilinear velocity and the number of motile sperm after 20 s acoustic exposure and without any adverse effects on sperm DNA integrity. It is noteworthy that traditional semen preparation methods (swim up and density gradient centrifugation) cause a comparable improvement in sperm motility (≈30% improvement),^[^ ^11^, ^77^, ^78^ ^]^ but by removing a sub‐population of immotile sperm. In comparison, our method offers a fundamental advantage, as we increase the swimming velocity and subsequently the motility of *the same* population of cells by exposing them to ultrasound, without the necessity for any preprocessing or selection. This improvement in sperm motility, in the absence of any chemical treatment and/or selection, provides new opportunities for selecting motile and viable sperm in clinics, to be used for the fertilization cycles either directly or after inducing sperm hypermotility using conventional clinical methods.^[^ ^73^ ^]^

The boosting effect was also observed for human sperm (Figure S3, Supporting Information) after exposure to ultrasound for 20 s using the optimum settings obtained for bull sperm (19.28 MHz and 2 W). An increase of 15% in VCL was achieved when human sperm were exposed to ultrasound, indicating the practicality of the method to also increase the swimming velocity and motility of human sperm. Increased sperm motility after treatment with extracellular ATP and prior to performing and IVF cycle is correlated with an increased fertilization rate.^[^ ^79^ ^]^ VCL is one of the key motility parameters that considerably affects the fertilization rate in IVF.^[^ ^80^ ^]^ ICSI as the other popular method in assisted reproduction, typically adopted for sperm samples with reduced concentration and motility. However, ICSI is an invasive procedure with potential detrimental effects on both the embryo and the offspring health.^[^ ^81^ ^]^ For samples with sufficient concentration but poor motility, this method may circumvent the need for ICSI by increasing sperm motility level or by rendering an immotile sperm motile to detect viability.

The work detailing the link between exposure conditions and increase in post‐exposure VCL has been limited to of bull sperm, sourced from two breeds (Holstein and Jersey) and treated homogenously. To demonstrate a broadened application of the technique, the boost effect in human sperm was also demonstrated, but using the optimum condition obtained for bull sperm. The result demonstrates a significant improvement of 15% on human sperm VCL post‐exposure, but further testing is required to optimize this effect for human sperm. In addition, like any type of artificial fertilization which circumvent the fundamental reproduction barriers,^[^ ^82^ ^]^ sperm which would be naturally discarded due to the lack of motility, could be potentially included in the fertilization process, in turn promoting the transmission of defective genetic traits. Therefore, future studies on whether IVF fertilization rates could be improved using sperm sample treated with ultrasound, as well as the genetic testing on the embryos fertilized with sperm treated with ultrasound are required as well.

Our findings offer the possibility of using high frequency ultrasound as a noninvasive and efficient method to increase both the number of motile sperm in the same sample and individual sperm motility. Therefore, combined with the traditional semen preparation method, this method could potentially improve the outcome of assisted reproductive technology. Moreover, our finding also provides new insight for altering the metabolism level of other microswimmers, such as *E. coli*, for applications relevant to environmental monitoring^[^ ^83^ ^]^ and protein production.^[^ ^84^ ^]^

## 4 Conclusion

We present a noninvasive approach to boost sperm motility utilizing the concept of acoustic based mechanotherapy. Treating bull sperm cells with high frequency ultrasound for a brief period (20 s) resulting in an increase of 34% in curvilinear velocity, 10% in linearity, and 32% in the number of motile sperm cells by rendering immotile sperm motile, without compromising cell viability (based on membrane integrity) and DNA integrity. Similarly, treating human sperm with the same setting, an increase of 15% in VCL has also been identified. This method offers promising opportunities to manage the male infertility issue by using ultrasound to induce/increase sperm motility and subsequently detect viable sperm for ICSI, circumventing the need for more invasive interventions.

## 5 Experimental Section

### Device Fabrication

The SAW devices operating at 19.28, 48.5, and 100 MHz comprised of two sets of IDTs, with eight, ten, and two finger pairs respectively (designed for optimum s11; matched at 50 Ω impedance). The IDTs were fabricated by applying a positive photoresist using standard UV‐photolithography on a 0.5 mm, single side polished, 128° Y‐cut, X‐propagating lithium niobate. Using e‐beam evaporation, the lithium niobate (LN) substrate was coated with 10 nm titanium adhesive layer, 160 nm gold conductive. Subsequently, the excess metal and photoresist was removed with the assistance of lift‐off technology. An additional 250 nm silicone dioxide layer was coated on top for protection. The master mold for the PDMS fluid chamber was 3D‐printed (Objet Eden 260 V) and soaked in 1 m NaOH solution for 1 h to remove the supporting materials, following which, a layer of silane (Sigma‐Aldrich, Missouri, USA) was assembled on the surface of the mold for easier detachment after PDMS casting. The premixed PDMS (SYLGARD 184, Dow Corning, with 1:5 mixing ratio of curing agent and polymer) was cast over the mold and degassed. Finally, it was placed on a 70 °C hotplate overnight to obtain the fully set PDMS fluid chamber.

### Sample Preparation

Cryogenically preserved bull semen sample (Holstein and Jersey) were purchased from ABS Global and were stored in 200 µL vials. Each sample used was thawed in 37 °C water bath for at least 5 min prior to extraction with an artificial insemination syringe. Fresh human sample were collected through masturbation into a sterile plastic cup after at least 2 d sex abstinence and were left for at least 30 min at 37 °C for liquefaction. This study was approved by the Monash University Human Research Ethics Committee. HEPES [2‐[4‐(2‐hydroxyethyl)piperazin‐1‐yl]ethanesulfonic acid)‐based salt buffered solution (NaHCO_3_ (4 × 10^−3^ m), KCL (5.3 × 10^−3^ m), NaCl (117 × 10^−3^ m), CaCl_2_ (2.3 × 10^−3^ m), Na_2_HPO_4_·2H_2_O (0.8 × 10^−3^ m), MgSO_4_ (0.8 × 10^−3^ m), phenol red (0.03 × 10^−3^ m), d‐glucose (5.5 × 10^−3^ m), Na pyruvate (0.33 × 10^−3^ m), and Na lactate (21.4 × 10^−3^ m)) supplemented with poly(vinyl alcohol) (1 mg mL^−1^) to prevent cell adhesion was mixed with the semen sample at a ratio of 10:1 for sperm motion analysis postultrasound exposure. To quantify sperm motility and vitality, 10 µL of propidium iodide and 10 µL of 50‐fold diluted SYBR14 in dimethyl sulfoxide (DMSO) (LIVE/DEAD Sperm Viability kit, ThermoFisher) were added to fluorescently label dead and live sperm respectively.

### Sperm Motility Analysis

The SAW device was mounted on a customized 3D printed microscope stage that includes an active cooling system. The input power on a peltier cooler was controlled with the aid of a temperature sensor to maintain the temperature of the microfluidic chip stable at 23–25 °C during the experiment, avoiding the heating effects associated with SAW actuation. A 35–40 µL of sperm sample was evenly distributed in the fluid chamber placed in between two IDTs. The IDT pair were actuated with a signal generator (BelektroniG F20 Power Saw, Freital, Germany) to generate a standing surface acoustic wave field. To quantify sperm motility, an image sequence of fluorescently labeled sperm motions at the same imaging field was recorded on chip before and after SAW generation with a 5‐MP C‐mount PixeLink camera (PL‐B872CU, Ottawa, Canada) equipped on an upright microscope (Olympus BX43, Tokyo, Japan) under green fluorescence light excited at the wavelength of 488 nm. The videos were recorded at 15 frames per second (15 fps) under the field of view of 1.4 mm × 1 mm. The recorded videos were then converted and processed in ImageJ with the OpenCASA plugin, where, only parameters of sperm tracked for 20–99 frames were included in the dataset. Sperm motility parameters were then reported as VCL, VAP, VSL, LIN, and motility percentage. Using this method, the motility of a bull sperm sample was measured four times, resulting in VCL values with no significant difference (Figure S6), indicating the reproducibility of the measurement and quantification method.

### Sperm Vitality Analysis

Postultrasound exposure at desired parameters, the fluid chamber was removed from the top surface of SAW device and fluorescently labeled sperm samples using sperm live/dead assay (ThermoFisher) was transferred into a hemocytometer (Paul Marienfeld Gmbh and Co. KG, Germany). The images under bright field, green fluorescence excited at the wavelength of 488 nm, and red fluorescence excited at the wavelength of 514 nm were captured and vitality was reported as the percentage of live sperm in total sperm population.

### Sperm DNA Integrity Analysis

Sperm DNA integrity is evaluated with sperm chromatin dispersion test using SpermFunc DNAf (BRED Life Science Technology Inc, Shenzhen, China). 60 µL of diluted semen sample with concentration from 5–10 million cells mL^−1^ was dispensed in the dissolved gel which was incubated at 80 °C for 20 min and balanced at 50 °C at least 5 min prior to utilization. 30 µL of the mixture of semen sample and gel was immediately dispensed on each well on the precoated slide was placed at the 2–8 °C fridge for at least 5 min until the mixture solidified. The slide was then immersed in solution A for 7 min exactly, incubated with solution B for 25 min under room temperature and horizontally into the tray filled with distilled water for 5 min. The slide was then vertically introduced to the slide barrels containing increasing concentration of ethanol (70%, 90%, and 100%) for 2 min. After air‐drying, 15–20 drops of Wright's stain were dispensed on the slide supplemented with 30–40 drops of the Wright's buffer for at least 30 min. At least 100 sperms were observed with a color camera (INFINITY3‐3UR, Lumenera) equipped on a general microscopy (Nikon Eclipse Ts2, Tokyo, Japan) under 40x magnification.

### Sperm Metabolic Activity Analysis

Postultrasound exposure at desired parameters, 10 µL of the sperm cells (out of 35–40 µL of sample) was carefully transferred from the device into a 96‐well‐plate containing 90 µL of media and 10 µL of Cell Counting Kit‐8 (CCK‐8, Sigma‐Aldrich) solution. The solution contains water soluble tetrazolium salt WST‐8 [2‐(2‐methoxy‐4‐nitrophenyl)‐3‐(4‐nitrophenyl)‐5‐(2,4‐disulfophenyl)‐2H‐tetrazolium, monosodium salt] that is converted into a formazan dye by living cells and therefore the change in the absorbance correlates with the metabolic activity when the number of cells is same in all samples. The cells were incubated immediately after ultrasound treatment in humidified incubator at 37 °C with 5% CO_2_ for 1 h. A second set of experiments was performed with sperm cells incubated in only media after ultrasound treatment for 30 min, followed by the addition of CCK‐8 solution and incubation for another 1 h. This is characterised as a negative control group, where the cells were not exposed to ultrasound. At the end of 1 h, the absorbance was recorded using a multiplate reader (Multiskan FC Microplate Photometer, Thermofisher Scientific) at 490 nm wavelength. Media which did not contain cells were used as blank for the absorbance calculations.

### Intracellular H_2_O_2_ Analysis

Intracellular H_2_O_2_ was analyzed using a fluorescent sensor (MAK164, Sigma‐Aldrich) with a green fluorescence excited at 490 nm and emission at 520 nm, according to the manufacturer`s instructions with slight modifications performed in order to analyze suspension cells. The sensor working solution was prepared by mixing fluorescent peroxide sensor solution with the assay buffer in the ration of 1 to 500 µL, respectively. Postultrasound exposure, 10 µL of the sperm cells (out of 35–40 µL of sample) was added to 100 µL of the sensor working solution and incubated at room temperature for 30 min. The sensor solution was removed by centrifugation (spinning for 5 min at 200 relative centrifugal force (rcf)) followed by the resuspension of cells in 20 µL of media. The cells were then transferred into a 96‐well plate and fixed by the addition of 80 µL of ice‐cold methanol at −20 °C. The fixed cells were washed with phosphate‐buffered saline for three times and imaged using a Nikon Eclipse Ts2 fluorescence microscope with CFI Plan Fluor 20 × /0.5 NA objective. A group of cells was incubated with 100 × 10^−6^ m of H_2_O_2_ in media for 30 min as positive control and the cells not exposed to SAW was labeled as negative control. The fluorescence was reported as the average fluorescence intensity (in the form of arbitrary units, a.u.)/cell acquired from four wells that contained at least 2000 cells in each group.

### Statistical Analysis

The set of individual sperm analyzed pre‐ and postexposure will differ as they swim in and out of the field of view and that each sample examined is inherently different prior to exposure. Postexposure sperm motility parameters (i.e., VCL, VAP, LIN, and motility percentage) and respective pre‐exposure parameters of each sperm analyzed were group‐mean centered to its corresponding pre‐exposure (i.e., direct control) quantity (Figures 2 and 3). Each sample was group‐mean centered at pre‐exposure levels to average their starting level comparable, providing a basis for fair comparison, accommodating for the heterogeneity in raw sample examined. Two‐way ANOVA with post hoc Bonferroni corrections was used to analyze the validity of exposure conditions by comparing the postexposure parameters with its pre‐exposure counterpart over the range of exposure conditions considered. Based on the minimum effect size of 0.24 captured as statistically significant (at 100 MHz, 750 mW, 10 s) using two‐way ANOVA (repeated measures, within‐between factors), at least 40 cells per each bull sperm sample were analyzed, higher than the minimum sample size of 34 required to detect the effect size of 0.24 at the significance level of 0.05 and with a 95% chance (accepted power). The performances across different exposure times were analyzed using the same statistical tests via comparing group‐mean centered postexposure motility parameters at each exposure condition.

Statistical analysis for sperm viability, DNA integrity, metabolic activity and intracellular H_2_O_2_ studies (Figures 4 and 5) were performed using One‐way ANOVA with post hoc Bonferroni corrections.

### Ethical Considerations

Permissions to conduct this study were obtained from Monash University Animal Ethics Committee and Monash University Human Research Ethics Committee.

## Conflict of Interest

The authors declare no conflict of interest.

## Authors Contribution

J.G., R.N., and A.N. designed the research, J.G. fabricated the device, and J.G. and E.D. performed the research. J.G., E.D., C.D., V.J.C., M.K.O., R.N., and A.N. analyzed the data and wrote the paper.

## Supporting information

Supporting Information

Supplemental Movie 1

Supplemental Movie 2

## Data Availability

The data that support the findings of this study are available from the corresponding author upon reasonable request.
