# Supplementary material for: High‐Frequency Ultrasound Boosts Bull and Human Sperm Motility
Source: Adv Sci (Weinh). 2022 Feb 9;9(11):2104362. doi: 10.1002/advs.202104362 (PMC9008414; doi:10.1002/advs.202104362)
Supplement: Supplementary file 1 — Supporting Information [file ADVS-9-2104362-s002.pdf]

WILEY-VCH

ADVANCED SCIENCE
Open Access

Supporting Information

for *Adv. Sci.*, DOI: 10.1002/advs.202104362

High-frequency ultrasound boosts bull and human sperm motility

Junyang Gai, Esma Dervisevic, Citsabehsan Devendran, Victor J. Cadarso, Moira K. O'Bryan, Reza Nosrati, and Adrian Neild**

Supporting Information

High-frequency ultrasound boosts sperm motility

Junyang Gai, Esma Dervisevic, Citsabehsan Devendran, Victor J. Cadarso,

Moira K. O'Bryan, Reza Nosrati, and Adrian Neild**

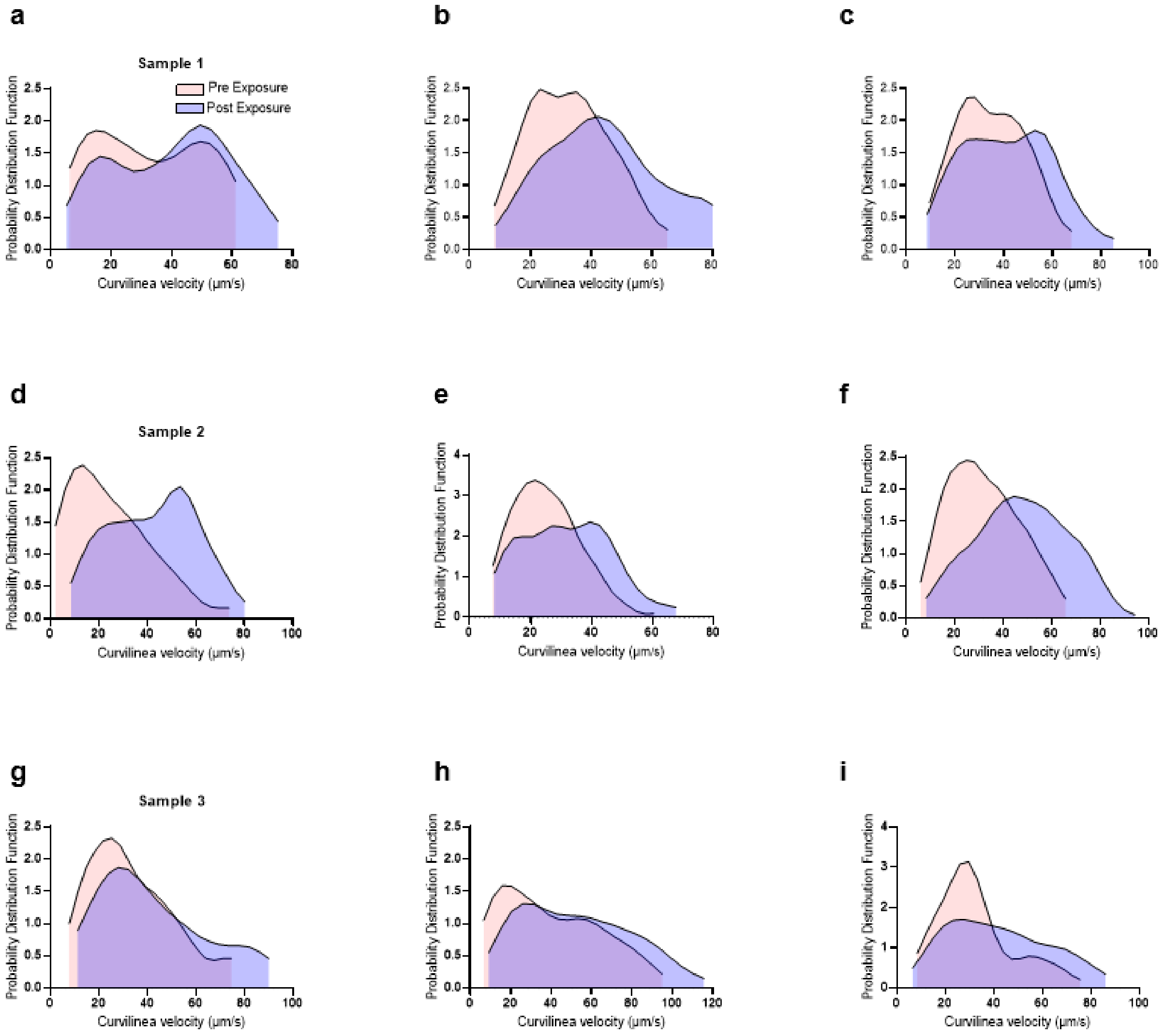

Fig. S1. Effect of SAW exposure time on curvilinear velocity (VCL) at the frequency of 19.28 MHz, power of 2 W, exposure time of 20 s. The probability distribution function (PDF) of the average curvilinear velocity for each tracked sperm in individual experiments tested for (**a**)-(**c**) sample1, (**d**)-(**f**) sample2, and (**g**)-(**i**) sample3. PDFs are obtained using the

kernel density estimation method. All data from 3 animals, 9 independent experiments with > 40 sperm analysed per experiment. Sperm trajectories are recorded for the same sample within the same imaging field pre- and post-exposure.

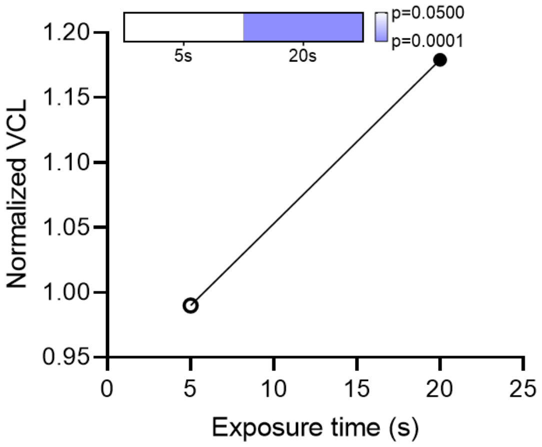

Fig. S2. Effect of SAW exposure time for sperm in buffer without Ca^{2+} on curvilinear velocity (VCL) at 19.28 MHz, 2 W. All data represented as grand mean from three independent experiments with > 40 sperm analysed per experiment. *p*-values of statistical analysis of normalized post-exposure VCL in comparison with pre-exposure VCL at each exposure condition, with hollow-shaped data point representing statistically non-significance ($p > 0.05$) and solid-shaped data point representing statistically significant ($p \leq 0.05$) data.

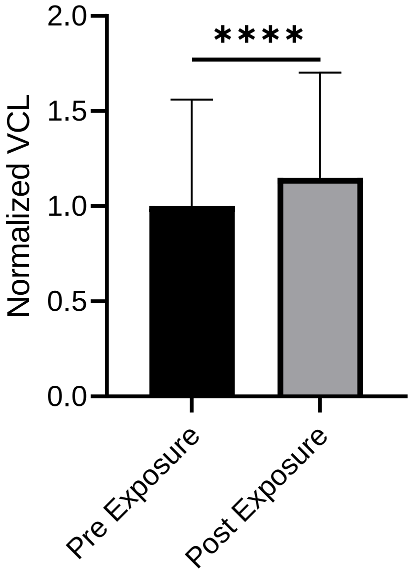

Fig. S3. **Effect of SAW exposure time for fresh human sperm on curvilinear velocity (VCL) at 19.28 MHz, 2 W, 20s.** All data represented as grand mean from three independent experiments peformed using different samples from three different donors. *p*-values were determined by t test with Welch's corrections. **** $p \leq 0.0001$.

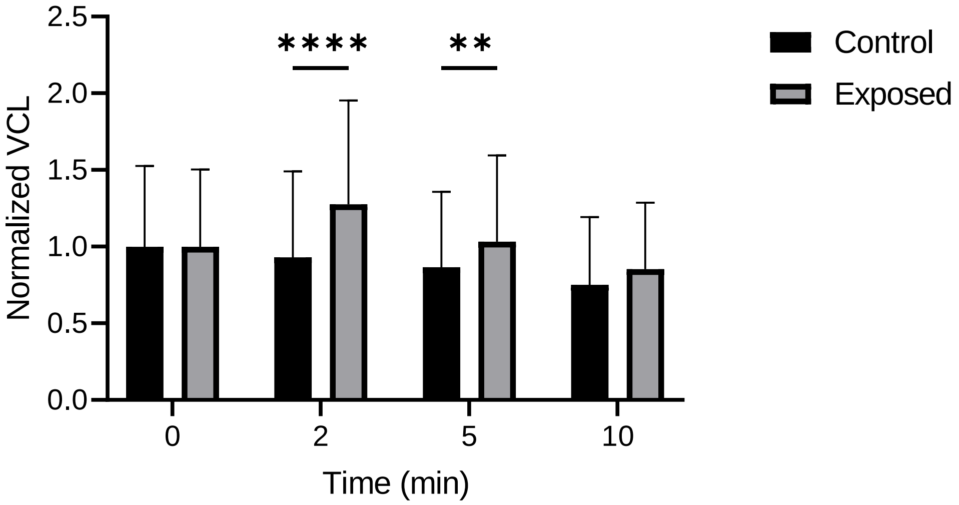

Fig. S4. Normalised curvilinear velocity (VCL) for bull sperm post-exposure (for 20 s at 19.28 MHz, 2 W) as a function of time after exposure as compared with a control group of unexposed cells. All data represented as grand mean from three independent experiments peformed using different samples from three different bulls. p-values were determined by two-way ANOVA test with Bonferroni corrections, ** $p \leq 0.01$, **** $p \leq 0.0001$.

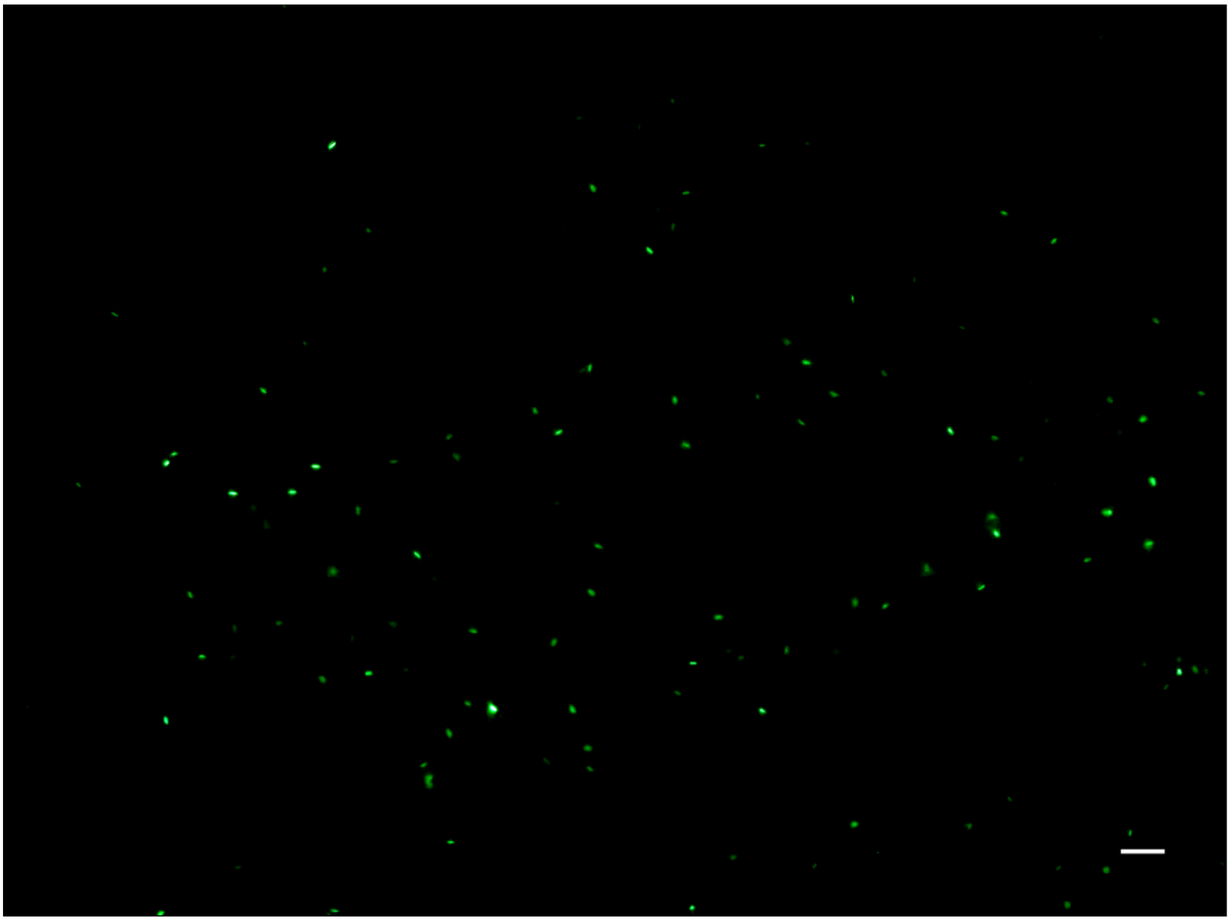

Fig. S5. A representative image of stained live sperm used for motility analysis. Live sperm were stained using SYBR 14 from theLIVE/DEAD™ Sperm Viability Kit and imaged at 15 frames per second for motility analysis. Scale bars, 50 µm.

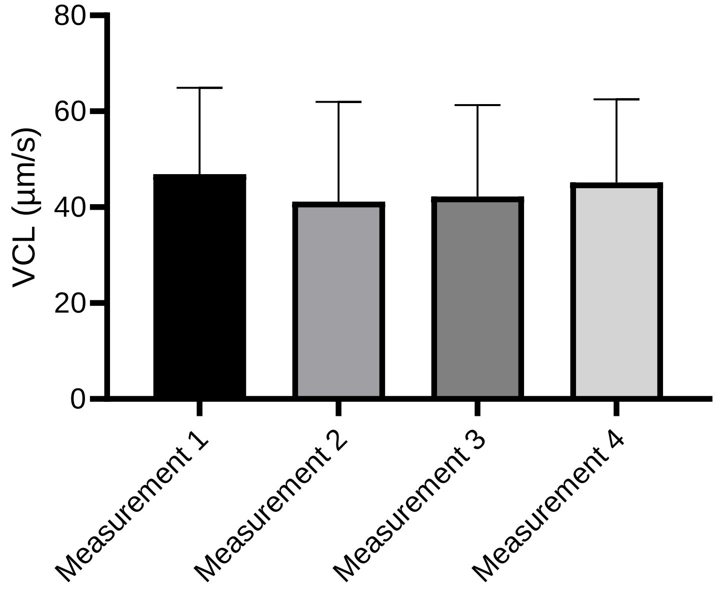

Fig. S6. Comparing the curvilinear velocity (VCL) of the same bull sperm sample between four different measurements. All data represented as mean ± s.d. from 4 independent measurements with > 40 sperm analyzed per experiment. No significant difference was observed for all data sets based on *p*-values that were determined using one-way ANOVA.

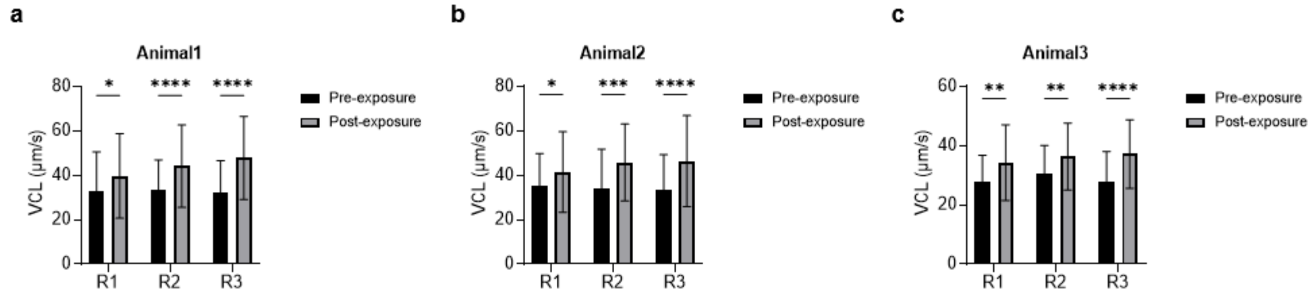

Figure S7. Sperm curvilinear velocity (VCL) when excited at 19.28 MHz, 2 W, and 20s. All data represented as mean ± SD from nine independent experiments peformed using triplicate samples (3 replicates per bull and 3 different bulls; n = 9), *p*-values were determined using two-way ANOVA test with post-hoc Bonferroni corrections, * $p \leq 0.05$, ** $p \leq 0.01$, *** $p \leq 0.001$, **** $p \leq 0.0001$.

19.28 MHz							
**Power**	**Exposure time**	**Pre-exposure**			**Post-exposure**		
		Mean	SD	N	Mean	SD	N
**250 mW**	5 s	1	0.435902	308	1.007342	0.393975	346
	10 s	1	0.433037	240	1.059541	0.414874	309
	15 s	1	0.440224	221	1.013945	0.370516	278
	2 0s	1	0.461408	184	1.098993	0.519808	205
	25 s	1	0.513908	218	1.114496	0.525561	247
**500 mW**	5 s	1	0.568412	157	0.977835	0.527622	251
	10 s	1	0.441894	230	1.095189	0.609139	154
	15 s	1	0.504765	273	1.080838	0.606078	139
	20 s	1	0.45092	288	1.044749	0.504077	353
	25 s	1	0.427338	188	1.118878	0.387483	256
**750 mW**	5 s	1	0.513493	159	1.010199	0.46153	256
	10 s	1	0.460143	129	1.05033	0.425183	163
	15 s	1	0.422631	136	1.027599	0.49981	168
	20 s	1	0.513856	156	1.198749	0.520838	256
	25 s	1	0.470849	166	1.143857	0.499542	256
**1 W**	5 s	1	0.545157	239	0.987498	0.563927	216
	10 s	1	0.573407	178	1.134909	0.578202	328
	15 s	1	0.579922	211	1.076674	0.567668	349
	20 s	1	0.649627	206	1.273791	0.741789	294
	25 s	1	0.574784	199	1.231138	0.697676	258
**2 W**	5 s	1	0.530346	170	1.081389	0.530147	283
	10 s	1	0.504842	233	1.082491	0.532956	258
	15 s	1	0.454704	186	1.155718	0.525271	430
	20 s	1	0.455292	415	1.34483	0.568764	609
	25 s	1	0.326781	191	1.262958	0.41698	291

Table S1. Normalized, standard deviation, and number analysed of pre- and post-exposure sperm curvilinear velocity (VCL) at 19.28 MHz. VCL of each sperm analysed in the same sample pre- and post-exposure were normalized with respect to the mean VCL of the sperms analysed in the sample pre-exposure.

48.5 MHz							
**Power**	**Exposure time**	**Pre-exposure**			**Post-exposure**		
		Mean	SD	N	Mean	SD	N
**250 mW**	**5 s**	1	0.353754	306	0.9872	0.334311	494
	**10 s**	1	0.423935	410	1.007985	0.387552	512
	**15 s**	1	0.412253	266	1.058307	0.425104	402
	**20 s**	1	0.366873	297	1.100591	0.36173	286
	**25 s**	1	0.391636	290	1.132536	0.31575	383
	**30 s**	1	0.428202	237	1.048643	0.357823	505
**500 mW**	**5 s**	1	0.453413	204	1.025454	0.439097	204
	**10 s**	1	0.436217	229	1.126177	0.442003	285
	**15 s**	1	0.401467	277	1.176063	0.334172	181
	**20 s**	1	0.403391	307	1.169607	0.407308	411
	**25 s**	1	0.434667	310	1.300834	0.392231	352
	**30 s**	1	0.438081	314	1.009429	0.434589	512
**750 mW**	**5 s**	1	0.497753	143	1.061551	0.477715	295
	**10 s**	1	0.474482	283	1.062013	0.466219	484
	**15 s**	1	0.392732	178	1.106572	0.379611	261
	**20 s**	1	0.490055	149	1.147683	0.519418	233
	**25 s**	1	0.433475	232	1.046194	0.458677	273
**1 W**	**5 s**	1	0.380413	190	1.053082	0.34764	294
	**10 s**	1	0.431401	165	1.155957	0.473275	238
	**15 s**	1	0.425402	172	1.234384	0.465874	178
	**20 s**	1	0.579416	171	1.078543	0.526011	262
	**25 s**	1	0.318213	203	1.066622	0.319171	170
**2 W**	**5 s**	1	0.442932	264	1.164182	0.40554	299
	**10 s**	1	0.569828	163	1.234903	0.453224	265
	**15 s**	1	0.335647	323	1.120252	0.370271	346
	**20 s**	1	0.541301	369	0.945073	0.486079	294
	**25 s**	1	0.458862	460	1.000108	0.441984	523

Table S2. Normalized, standard deviation, and number analysed of pre- and post-exposure sperm curvilinear velocity (VCL) at 48.5 MHz. VCL of each sperm analysed in the same sample pre- and post-exposure were normalized with respect to the mean VCL of the sperms analysed in the sample pre-exposure.

100 MHz							
**Power**	**Exposure time**	**Pre-exposure**			**Post-exposure**		
		Mean	SD	N	Mean	SD	N
**250 mW**	**5 s**	1	0.438349	292	1.035148	0.465195	446
	**10 s**	1	0.400112	174	1.042419	0.399232	329
	**15 s**	1	0.462685	157	1.079628	0.43763	339
	**2 0s**	1	0.587319	303	1.036075	0.530962	374
	**25 s**	1	0.381862	205	0.985145	0.380576	366
**500 mW**	**5 s**	1	0.512872	312	1.068896	0.471745	549
	**10 s**	1	0.642841	270	1.12891	0.615431	410
	**15 s**	1	0.595826	283	1.154836	0.610524	330
	**20 s**	1	0.587319	303	1.036075	0.530962	374
	**25 s**	1	0.583413	253	1.052516	0.484261	411
**750 mW**	**5 s**	1	0.481897	226	1.12258	0.424901	383
	**10 s**	1	0.543472	151	1.124498	0.49674	269
	**15 s**	1	0.573133	154	1.237371	0.514734	342
	**20 s**	1	0.460007	188	1.049246	0.457968	419
	**25 s**	1	0.420873	249	1.010011	0.416476	471
**1 W**	**5 s**	1	0.483941	256	1.148611	0.447836	365
	**10 s**	1	0.554708	211	1.254539	0.647106	267
	**15 s**	1	0.516765	223	1.267411	0.527577	367
	**20 s**	1	0.441847	306	1.046877	0.418555	463
	**25 s**	1	0.369275	208	1.062457	0.37271	339
**2 W**	**5 s**	1	0.369588	265	1.018419	0.384311	503
	**10 s**	1	0.407772	232	1.079255	0.422003	420
	**15 s**	1	0.372592	323	1.03472	0.384483	368
	**20 s**	1	0.407807	284	1.055614	0.439767	308
	**25 s**	1	0.498431	298	1.032368	0.543448	500

Table S3. Normalized, standard deviation, and number analysed of pre- and post-exposure sperm curvilinear velocity (VCL) at 100 MHz. VCL of each sperm analysed in the same sample pre- and post-exposure were normalized with respect to the mean VCL of the sperms analysed in the sample pre-exposure.

19.28 MHz					
Time	Power				
	250 mW	500 mW	750 mW	1 W	2 W
**5 s**	ns	ns	ns	ns	ns
**10 s**	ns	ns	ns	ns	ns
**15 s**	ns	ns	ns	ns	*
**20 s**	ns	ns	***	****	****
**25 s**	ns	*	*	***	****

Table S4. *p* values of statistical analysis of normalized post-exposure VCL in comparison with pre-exposure VCL at each exposure condition at 19.28 MHz. VCL of each sperm analysed in the same sample pre- and post-exposure were normalized with respect to the mean VCL of the sperms analysed in the sample pre-exposure.

48.5 MHz					
Time	Power				
	250 mW	500 mW	750 mW	1 W	2 W
**5 s**	ns	ns	ns	ns	****
**10 s**	ns	**	*	**	****
**15 s**	ns	****	*	****	**
**20 s**	**	****	*	ns	ns
**25 s**	****	****	ns	ns	ns
**30 s**	ns	ns			

Table S5. *p* values of statistical analysis of normalized post-exposure VCL in comparison with pre-exposure VCL at each exposure condition at 48.5 MHz. VCL of each sperm analysed in the same sample pre- and post-exposure were normalized with respect to the mean VCL of the sperms analysed in the sample pre-exposure.

100 MHz					
Time	Power				
	250 mW	500 mW	750 mW	1 W	2 W
**5 s**	ns	ns	**	***	ns
**10 s**	ns	*	*	****	ns
**15 s**	ns	*	****	****	ns
**20 s**	ns	ns	ns	ns	ns
**25 s**	ns	ns	ns	ns	ns

Table S6. *p* values of statistical analysis of normalized post-exposure VCL in comparison with pre-exposure VCL at each exposure condition at 100 MHz. VCL of each sperm analysed in the same sample pre- and post-exposure were normalized with respect to the mean VCL of the sperms analysed in the sample pre-exposure.

19.28MHz							
**Power**	**Exposure time**	**Number of cells (pre-exposure)**			**Number of cells (post-exposure)**		
		**Sample1**	**Sample2**	**Sample3**	**Sample1**	**Sample2**	**Sample3**
**250mW**	5s	94	145	70	136	74	134
	10s	109	53	78	131	77	101
	15s	78	90	53	85	73	120
	20s	58	90	44	79	49	77
	25s	48	50	120	102	83	62
**500mW**	5s	54	46	57	87	81	83
	10s	46	121	63	62	63	52
	15s	126	101	46	52	41	62
	20s	56	150	82	157	57	139
	25s	40	63	85	82	89	95
**750mW**	5s	66	44	49	108	83	83
	10s	47	40	42	57	49	57
	15s	45	71	42	50	40	84
	20s	42	68	64	71	155	169
	25s	43	46	78	100	59	133
**1W**	5s	62	107	70	105	70	41
	10s	67	55	56	96	100	132
	15s	86	51	74	107	100	142
	20s	71	58	77	111	89	94
	25s	54	65	80	131	42	85
**2W**	5s	56	59	55	138	67	78
	10s	81	71	81	98	125	89
	15s	80	89	47	135	156	139
	20s	66	117	232	120	219	270
	25s	61	79	51	76	88	127

Table S7. Number of cells analysed pre- and post-exposure sperm curvilinear velocity (VCL) for each sample at 19.28 MHz. VCL of each sperm analysed in the same sample pre- and post-exposure were normalized with respect to the mean VCL of the sperms analysed in the sample pre-exposure.

48.5MHz							
**Power**	**Exposure time**	**Number of cells (pre-exposure)**			**Number of cells (post-exposure)**		
		**Sample1**	**Sample2**	**Sample3**	**Sample1**	**Sample2**	**Sample3**
**250mW**	**5s**	74	140	92	79	139	276
	**10s**	117	141	152	133	182	272
	**15s**	62	64	140	198	118	86
	**20s**	130	104	63	172	50	64
	**25s**	144	67	79	99	97	187
	**30s**	77	81	79	152	179	174
**500mW**	**5s**	84	71	49	82	48	74
	**10s**	59	68	102	84	139	62
	**15s**	51	172	54	47	81	53
	**20s**	62	162	83	63	145	203
	**25s**	156	112	42	70	222	60
**750mW**	**5s**	49	44	50	103	114	78
	**10s**	101	76	106	139	93	252
	**15s**	70	58	50	90	75	96
	**20s**	57	47	45	74	108	51
	**25s**	44	140	48	47	144	82
**1W**	**5s**	49	89	52	69	146	79
	**10s**	51	44	70	87	66	85
	**15s**	44	62	66	43	88	47
	**20s**	61	40	70	84	79	99
	**25s**	82	81	40	73	56	46
**2W**	**5s**	57	113	94	77	73	149
	**10s**	96	67	132	67	125	73
	**15s**	117	77	129	37	94	133
	**20s**	156	109	104	57	123	114
	**25s**	142	143	175	102	155	266

Table S8. Number of cells analysed pre- and post-exposure sperm curvilinear velocity (VCL) for each sample at 48.5 MHz. VCL of each sperm analysed in the same sample pre- and post-exposure were normalized with respect to the mean VCL of the sperms analysed in the sample pre-exposure.

100MHz							
**Power**	**Exposure time**	**Number of cells (pre-exposure)**			**Number of cells (post-exposure)**		
		**Sample1**	**Sample2**	**Sample3**	**Sample1**	**Sample2**	**Sample3**
**250mW**	**5s**	65	71	157	91	137	218
	**10s**	77	49	48	86	129	114
	**15s**	44	49	64	159	73	107
	**20s**	106	112	85	134	106	136
	**25s**	76	89	41	152	137	77
**500mW**	**5s**	87	81	144	216	181	152
	**10s**	118	101	51	154	144	112
	**15s**	117	57	109	96	94	140
	**20s**	106	112	85	134	104	136
	**25s**	133	54	66	180	108	123
**750mW**	**5s**	47	72	107	94	119	170
	**10s**	40	44	67	58	100	111
	**15s**	43	44	67	131	40	84
	**20s**	44	47	97	59	184	176
	**25s**	99	60	90	202	146	123
**1W**	**5s**	93	90	73	108	160	97
	**10s**	62	68	81	80	89	78
	**15s**	67	63	93	114	83	170
	**20s**	83	107	116	115	169	179
	**25s**	89	119	181	163	176	292
**2W**	**5s**	84	76	105	176	162	165
	**10s**	50	92	90	133	169	118
	**15s**	138	94	92	137	147	84
	**20s**	96	124	64	60	151	97
	**25s**	89	142	68	174	219	107

Table S9. Number of cells analysed pre- and post-exposure sperm curvilinear velocity (VCL) for each sample at 100 MHz. VCL of each sperm analysed in the same sample pre- and post-exposure were normalized with respect to the mean VCL of the sperms analysed in the sample pre-exposure.

Movie S1. Single sperm swimming velocity increased post exposure. The video is in real time, and scale bars, 50 µm.

Movie S2. Single immotile sperm turned motile post exposure. The video is in real time, and scale bars, 50 µm.
